# Perception of Dog Welfare in Veterinary Students: A Six-Year Study of Ethical Priorities, Cohort Variation, and Influencing Factors

**DOI:** 10.3390/ani16091385

**Published:** 2026-05-01

**Authors:** Luis Alberto Henríquez-Hernández, Beatriz Martín-Cruz, Octavio P. Luzardo, Manuel Zumbado

**Affiliations:** Legal Veterinary and Deontology, Clinical Sciences Department, Universidad de Las Palmas de Gran Canaria, Paseo Blas Cabrera Felipe s/n, 35016 Las Palmas de Gran Canaria, Spain; beatriz.martin@ulpgc.es (B.M.-C.); octavio.perez@ulpgc.es (O.P.L.); manuel.zumbado@ulpgc.es (M.Z.)

**Keywords:** animal welfare, veterinary education, veterinary students, welfare perception, repeated cross-sectional study

## Abstract

Veterinary students’ perceptions of animal welfare are shaped by both educational exposure and individual background, with direct implications for future clinical decision-making. This study evaluated 157 ninth-semester veterinary students enrolled in a Deontology and Veterinary Legal course at the University of Las Palmas de Gran Canaria (Spain) across six academic years (2019/2020–2025/2026), excluding 2020/2021 due to pandemic-related teaching modifications. Participants completed a structured, previously published questionnaire assessing twelve common dog welfare issues on a 5-point Likert scale, subsequently grouped into five dog welfare dimensions. Students consistently prioritized overt and severe dog welfare concerns, including abuse or active cruelty, lack of treatment to prevent suffering, and malnutrition, while assigning lower importance to breed-related conditions and behavioral problems. Significant differences across academic years were identified for seven items and all dimensions, revealing temporal variability in dog welfare perception between cohorts. Gender and student background also influenced responses, with female and exchange students generally assigning higher scores to selected issues. These findings suggest that the perception of dog welfare is not static but varies between cohorts across academic years and is shaped by sociocultural factors. Strengthening veterinary curricula to address less visible and socially normalized dog welfare problems may be critical to ensuring comprehensive and ethically grounded professional practice.

## Simple Summary

Veterinarians play a key role in protecting animal welfare, and their ability to identify and prioritize welfare problems begins during their university training. This study explored how veterinary students perceive different dog welfare issues and whether these perceptions differ between cohorts across time. We surveyed 157 students in their final year of a veterinary degree over six academic years using a questionnaire that assessed the importance of twelve common problems in dogs. Students consistently rated obvious and severe problems, such as abuse, lack of treatment, and malnutrition, as highly important. In contrast, less visible or more complex issues, such as breed-related conditions and behavioral problems, were considered less important. Differences between academic years suggest that students’ views on dog welfare vary between cohorts. In addition, female students and those from different geographical backgrounds tended to assign higher importance to some dog welfare issues. These findings highlight that not all dog welfare problems are equally recognized by future veterinarians. Improving veterinary education to better address less visible and socially accepted welfare issues may help ensure that future professionals are better prepared to make balanced and ethical decisions in clinical practice.

## 1. Introduction

Veterinarians are widely recognized as key professionals responsible for safeguarding animal welfare, a role that extends beyond the diagnosis and treatment of disease to encompass animals’ physical, mental, and behavioral well-being [1]. Across diverse contexts—including companion animals, livestock production systems, laboratory settings, and wildlife conservation—veterinarians are expected to integrate scientific knowledge with ethical responsibility in order to prevent suffering and promote positive welfare states [2,3]. International organizations such as the World Organization for Animal Health (OIE), the American Veterinary Medical Association (AVMA), the Federation of Veterinarians of Europe (FVE), and the World Small Animal Veterinary Association (WSAVA) explicitly position veterinarians as primary advocates and custodians of animal welfare [4]. This professional mandate is further reinforced by legal frameworks and codes of conduct, which increasingly assign veterinarians a duty of care not only toward animals, but also toward clients and society [5]. Consequently, decision-making in veterinary practice is inherently ethical, requiring practitioners to balance clinical, social, and moral considerations. In this context, animal welfare cannot be understood solely as a technical or biomedical issue, but as a fundamentally ethical domain embedded within veterinary professional identity [6].

Building on this professional responsibility, animal welfare is increasingly understood as a multidimensional construct that extends beyond the traditional framework of the Five Freedoms [7]. Contemporary approaches emphasize that welfare encompasses not only physical health, but also behavioral and mental states, as well as the environmental conditions in which animals live [8,9]. This broader perspective highlights that different welfare issues vary in their visibility and immediacy: while conditions such as acute pain or malnutrition are readily recognized, others—such as chronic stress, lack of social interaction, or insufficient mental stimulation—may be subtler and therefore underestimated [10,11,12]. Importantly, the assessment of dog welfare is often context-dependent, as dogs may display markedly different behavioral and physiological responses in clinical environments compared to their home or habitual settings [13,14]. This variability can influence both the identification of welfare problems and their perceived severity, adding an additional layer of complexity to veterinary decision-making [15]. Furthermore, distinctions can be drawn between active forms of harm, such as abuse or cruelty, and more passive forms of welfare compromise, including neglect, inadequate housing, or insufficient preventive care [16,17,18]. These differences are not merely theoretical, but may influence how welfare problems are perceived and prioritized in practice. Consequently, not all dog welfare issues are equally recognized or valued, even among trained or future veterinary professionals.

Previous research has explored how different stakeholders perceive animal welfare issues, revealing important variability in the prioritization of specific concerns. Early work in veterinary practitioners identified a distinction between frequently encountered problems and those considered most severe, with issues such as lack of treatment for suffering, abuse, and malnutrition consistently rated as highly important, while others, including obesity and breed-related conditions, were perceived differently depending on context [19]. Similar patterns have been observed in studies involving veterinary students and professionals, where overt forms of suffering—such as physical abuse—tend to be consistently prioritized, whereas more subtle or multifactorial conditions, including behavioral problems, lack of social interaction, or breed-associated disorders, show greater variability in perceived importance [20]. Moreover, factors such as level of training, professional experience, and specialization appear to influence sensitivity to less visible dog welfare issues, with more specialized individuals often assigning greater importance to behavioral and emotional aspects [20]. Emerging evidence also highlights that certain welfare problems, such as obesity or altered feeding behaviors, may be normalized or underestimated despite their clinical relevance [21]. Overall, these findings suggest that the perception of dog welfare issues is not uniform, but shaped by experience, knowledge, and contextual factors.

Beyond these descriptive findings, the perception of animal welfare issues is increasingly recognized as a complex and constructed phenomenon, shaped by educational, psychological, and sociocultural factors. Veterinary training plays a central role in this process, not only by providing knowledge, but also by exposing students to ethical dilemmas and contested welfare issues that require critical evaluation and discussion [22]. At the same time, individual and sociodemographic characteristics—such as gender, background, or prior experience with animals—have been shown to influence sensitivity to animal welfare and abuse, highlighting the subjective component underlying these assessments [23,24]. From a psychological perspective, perception is mediated by values, attitudes, and social norms, which may condition how different forms of suffering are recognized and prioritized. Furthermore, the professional responsibility of veterinarians to identify and respond to animal abuse situates welfare perception within a broader ethical and public health framework, where recognition of subtle or ambiguous cases becomes essential [25]. Taken together, these factors suggest that the perception of animal welfare is not merely acquired through formal education, but actively constructed through the interaction of knowledge, experience, and social context, potentially leading to variability across academic cohorts and student backgrounds.

Despite the growing body of research on the perception of animal welfare, important gaps remain. Most existing studies are cross-sectional and focus on single cohorts or professional groups, limiting the understanding of how ethical sensitivity toward dog welfare issues may evolve throughout training or vary across different student backgrounds. In this context, educational settings provide a valuable opportunity to explore these dynamics. The present study was conducted within a voluntary activity embedded in a Deontology and Veterinary Legal course taught in the ninth semester of the Veterinary Medicine degree at the University of Las Palmas de Gran Canaria (Spain). Using a structured questionnaire adapted from previous research [19,20], students were asked to evaluate the importance of twelve issues affecting dog welfare. The aim of this study was to assess students’ perception of these welfare issues over six academic years, and to explore potential differences across academic cohorts and demographic variables.

## 2. Materials and Methods

### 2.1. Sample

The study population consisted of veterinary students enrolled in the ninth semester of the Deontology and Veterinary Legal course at the University of Las Palmas de Gran Canaria (Spain), spanning six academic years: 2019/2020, 2021/2022, 2022/2023, 2023/2024, 2024/2025, and 2025/2026. Data for the 2020/2021 academic year were not collected due to modifications in course delivery required by the global public health alert. Participation in the survey was voluntary, although it contributed to the continuous assessment component of the course. All responses were treated anonymously, and no formal ethical approval was required, as the activity was an integral part of the course curriculum and did not involve any intervention beyond the normal educational context.

Demographic information on age, sex, and place of origin was obtained from the university’s administrative records. As a result, detailed information on the specific origin of exchange students was not available, and these participants were included in the “other” category for the variable of origin. Students were classified as living in an urban home setting if they resided in any of the capital cities of the Canary Islands; all other locations were considered rural or not available for exchange students.

### 2.2. Questionnaire

Data were collected using a structured questionnaire adapted from Yeates and Main (2011) [19] and subsequently applied in Spanish by Luño et al. (2017) [20]. The instrument consisted of twelve items assessing the importance of common dog welfare issues, translated and adapted into Spanish following Luño’s design to maintain consistency with previous studies. The items included: (1) Obesity, (2) Chronic pain or poor mobility, (3) Breed-related conditions, (4) Behavioral problems, (5) Lack of treatment, including euthanasia, to prevent suffering, (6) Lack of sufficient exercise or space, (7) Lack of mental stimulation, (8) Lack of routine preventive veterinary care, (9) Abuse or active cruelty, (10) Malnutrition, (11) Lack of sufficient company, and (12) Lack of shelter. Each item was rated on a 5-point Likert scale, ranging from 0 (“not important”) to 4 (“very important”).

### 2.3. Data Collection and Statistical Analysis

The questionnaire remained open throughout the entire academic term and was hosted on the course’s virtual campus, allowing students to complete it at their convenience.

Likert-scale survey responses were treated as categorical variables for statistical analysis to avoid assuming equal intervals between response categories. This approach, which has been employed previously [26,27], allows for the examination of the distribution of responses across all categories without imposing parametric assumptions.

Descriptive statistics were calculated for all items and dimensions, including mean, standard deviation, and median. Comparisons between academic years and other categorical variables were performed using non-parametric tests, including the Kruskal–Wallis test for multiple-group comparisons and the Mann–Whitney U test for pairwise analyses where appropriate. Correlations between continuous variables (e.g., age) and dog welfare scores were assessed using Spearman’s rank correlation coefficient.

To facilitate analysis and interpretation, the twelve items were aggregated into five dimensions reflecting broader aspects of dog welfare as follows: Physical welfare, sum of items 2 (Chronic pain or poor mobility) and 10 (Malnutrition); Mental welfare, sum of items 4 (Behavioral problems), 7 (Lack of mental stimulation), and 11 (Lack of sufficient company); Environmental structural welfare, sum of items 6 (Lack of sufficient exercise or space) and 12 (Lack of shelter); Socially normalized issues, sum of items 1 (Obesity) and 3 (Breed-related conditions); and Malpractice, sum of items 5 (Lack of treatment, including euthanasia, to prevent suffering), 8 (Lack of routine preventive veterinary care), and 9 (Abuse or active cruelty). A total score was also calculated as the sum of all dimensions, providing a composite measure of the relevant dog welfare domain. Importantly, these dimensions were constructed as a theoretical aggregation of conceptually related items rather than as empirically derived latent constructs, as the original questionnaire was designed to assess discrete dog welfare concerns at the item level. Therefore, these groupings should be interpreted as analytical summaries intended to facilitate interpretation of broader patterns, rather than as psychometric scales. Given the heterogeneity of the items and their potential conceptual overlap across domains, internal consistency indices (e.g., Cronbach’s alpha) were not calculated. This approach allowed the identification of broader patterns in students’ perception of dog welfare, while preserving the specificity of individual welfare issues.

An alpha level of 0.05 was set as the threshold for statistical significance in all analyses. For database management and statistical analysis, the PASW Statistics software (version 19.0, SPSS Inc., Chicago, IL, USA) was used.

## 3. Results

A total of 157 students responded to the survey, including 119 women (75.8%) and 38 men (24.2%), with a mean age of 24.6 ± 3.1 years. Most participants were from Gran Canaria island (*n* = 101, 66.4%) and reported living in rural areas (52.7%). Response rates by academic year ranged from 18.5% of enrolled students in 2019/2020 to 66.1% in 2024/2025. Detailed demographic characteristics by academic year are presented in Supplementary Table S1.

The distribution of responses for each dog welfare issue across the entire sample is presented in Supplementary Table S2. Overall, all items received moderate to high importance scores, although notable variability was observed between issues. The highest-rated item was abuse or active cruelty, with a mean score of 4.0 ± 0.3, and 157 respondents (98.7%) assigning the maximum score. In contrast, breed-related conditions received the lowest mean score (2.5 ± 0.9), with only 24 participants (15.3%) rating it as highly important. Bivariate correlations between the twelve dog welfare issues revealed a consistent pattern of positive associations, with all correlations reaching statistical significance (*p* < 0.05). This indicates that higher importance assigned to one dog welfare issue tended to be associated with higher ratings for others. The strongest correlation was observed between *obesity* and *breed-related conditions* (Pearson’s r = 0.448, *p* < 0.001), whereas the weakest association was found between *chronic pain or poor mobility* and *malnutrition* (Pearson’s r = 0.164, *p* = 0.040).

### 3.1. Cross-Sectional Comparisons by Academic Year

**Figure 1:**
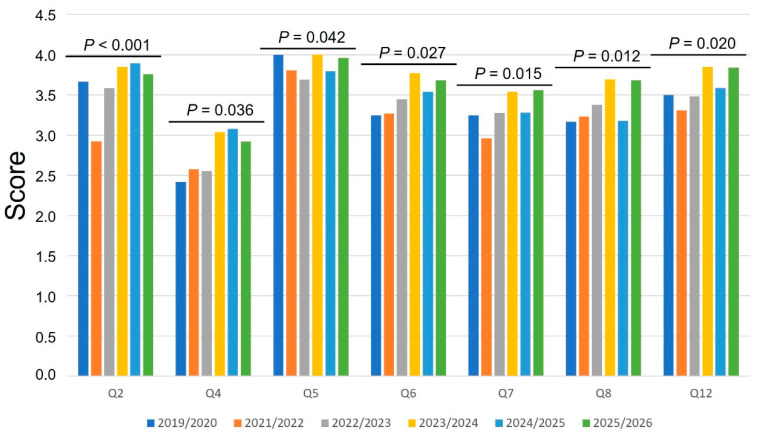
Temporal variation in veterinary students’ perception of the importance of selected dog welfare issues across six academic years (2019/2020–2025/2026). Only items showing statistically significant differences between cohorts are presented: Q2, chronic pain or poor mobility; Q4, behavioral problems; Q5, lack of treatment, including euthanasia for suffering; Q6, lack of sufficient exercise or space; Q7, lack of sufficient mental stimulation; Q8, lack of routine preventive veterinary care; and Q12, lack of shelter. Bars represent mean scores, reflecting the relative importance assigned to each dog welfare issue (higher scores indicate greater perceived importance). Exact *p*-values are indicated above the corresponding groups (Kruskal–Wallis test).

Temporal variation in the perceived importance of selected dog welfare issues across academic years is presented in Figure 1. Statistically significant differences between cohorts were observed for seven of the twelve items: chronic pain or poor mobility (Q2; *p* < 0.001), behavioral problems (Q4; *p* = 0.036), lack of treatment to prevent suffering (Q5; *p* = 0.042), lack of sufficient exercise or space (Q6; *p* = 0.027), lack of mental stimulation (Q7; *p* = 0.015), lack of preventive veterinary care (Q8; *p* = 0.012), and lack of shelter (Q12; *p* = 0.020). However, cross-sectional differences should be interpreted with caution, as academic cohorts may differ not only temporally but also in their demographic composition, which was not controlled for across years.

Across these items, mean scores fluctuated between academic years without a clear linear trend. For instance, chronic pain or poor mobility (Q2) showed a marked decrease in 2021/2022 (mean = 2.9) compared to 2019/2020 (3.7), followed by a progressive increase in subsequent cohorts, reaching the highest values in 2024/2025 (3.9). A similar pattern of variability was observed for behavioral problems (Q4), with relatively stable scores in earlier years and higher values in more recent cohorts. Items related to resource provision and preventive care, such as lack of sufficient exercise or space (Q6), lack of mental stimulation (Q7), and lack of preventive veterinary care (Q8), also showed moderate fluctuations, generally tending toward higher scores in later academic years, although this pattern was not consistent across all cohorts. In contrast, lack of treatment to prevent suffering (Q5) remained consistently highly rated across all years, with mean scores close to the maximum value.

**Figure 2:**
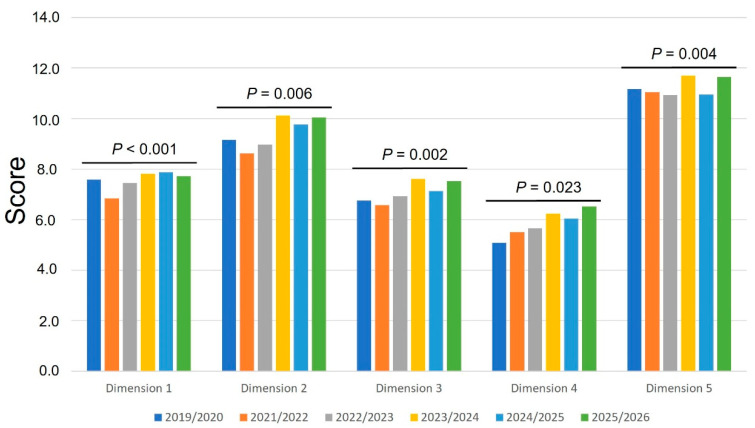
Temporal variation in veterinary students’ perception of the importance of selected dog welfare dimensions across six academic years (2019/2020–2025/2026). Dimension 1: Physical welfare (Sum of items 2 (Chronic pain or limited mobility) and 10 (Malnutrition)); dimension 2: Mental welfare (Sum of items 4 (Behavioral problems), 7 (Lack of sufficient mental stimulation), and 11 (Lack of company)); dimension 3: Environmental structural welfare (Sum of items 6 (Lack of sufficient exercise or space) and 12 (Lack of shelter)); dimension 4: Socially normalized issues (Sum of items 1 (Obesity) and 3 (Breed-related conditions)); and dimension 5: Malpractice (Sum of items 5 (Lack of treatment to avoid suffering (including euthanasia), 8 (Lack of routine preventive veterinary care) and 9 (Abuse or active cruelty)). Bars represent mean scores, reflecting the relative importance assigned to each dog welfare dimension (higher scores indicate greater perceived importance). Exact *p*-values are indicated above the corresponding groups (Kruskal–Wallis test).

In addition to individual items, all five composite dimensions showed statistically significant differences across academic years (Figure 2). These included physical dog welfare (Dimension 1; *p* < 0.001), mental dog welfare (Dimension 2; *p* = 0.006), environmental structural dog welfare (Dimension 3; *p* = 0.002), socially normalized issues (Dimension 4; *p* = 0.023), and malpractice (Dimension 5; *p* = 0.004). Across dimensions, mean scores exhibited variability between cohorts without a consistent linear trend. Physical dog welfare (Dimension 1) showed a decrease in 2021/2022 (mean = 6.8) compared to 2019/2020 (7.6), followed by a progressive increase in subsequent years. A similar pattern was observed for mental dog welfare (Dimension 2), which reached its highest values in the most recent cohorts (2023/2024 = 10.1 out of 12 points, and 2025/2026 = 10.0 out of 12 points), and the lowest score in the academic year 2021/2022 (8.6 out of 12 points). Dimensions related to environmental and management factors, such as environmental structural dog welfare (Dimension 3), also showed moderate increases in later years, although with some fluctuations. Notably, socially normalized issues (Dimension 4), including obesity and breed-related conditions, exhibited a gradual increase over time, reaching the highest mean value in 2025/2026 (6.5 out of 8 points). In contrast, malpractice (Dimension 5) remained consistently high across all cohorts, with mean scores close to the upper limit of the scale.

The total score, calculated as the sum of all dimensions, also differed significantly between academic years (*p* < 0.001), showing a similar pattern of variability with higher values in more recent cohorts (more than 40 out of 48 points for the academic years 2023/2024, 2024/2025 and 2025/2026).

### 3.2. Influence of Demographic Variables

Associations between demographic variables and students’ perception of dog welfare issues are presented in Table 1. No statistically significant differences were observed in relation to gender, home setting (urban vs. rural), or age. Significant differences were identified according to place of origin for several dog welfare issues. Exchange students with unavailable origin, classified within the “other” category, consistently assigned higher importance scores compared to those from Gran Canaria and Tenerife. This was observed for breed-related conditions (3.0 ± 0.9 vs. 2.5 ± 0.9 and 2.3 ± 0.9, respectively; *p* = 0.038), behavioral problems (3.2 ± 0.7 vs. 2.8 ± 0.9 and 2.6 ± 1.1; *p* = 0.033), lack of sufficient exercise or space (3.8 ± 0.5 vs. 3.4 ± 0.6 and 3.6 ± 0.7; *p* = 0.010), lack of sufficient company (3.7 ± 0.5 vs. 3.3 ± 0.7 and 3.3 ± 0.9; *p* = 0.026), and lack of shelter (3.9 ± 0.3 vs. 3.5 ± 0.7 and 3.7 ± 0.9; *p* = 0.017).

Analyses at the dimensional level are presented in Table 2. No statistically significant associations were found between age or home setting and any of the composite dimensions. With respect to gender, a significant difference was observed for environmental structural dog welfare, with female students assigning higher scores than male students (7.2 ± 1.1 vs. 6.8 ± 1.1; *p* = 0.037). No other dimensions showed significant gender-related differences. Regarding place of origin, several significant associations were identified. Students in the “other” category reported higher scores for mental dog welfare (10.6 ± 1.3 vs. 9.3 ± 1.7 and 9.1 ± 2.4; *p* = 0.005) and environmental structural dog welfare (7.7 ± 0.6 vs. 6.9 ± 1.1 and 7.3 ± 1.5; *p* = 0.002) compared to those from Gran Canaria and Tenerife. Similarly, the total score differed significantly across origin groups, with the highest values observed in the “other” category (44.0 ± 2.9 vs. 41.0 ± 4.3 and 40.1 ± 8.2; *p* = 0.008). No statistically significant differences were observed in relation to age and home setting.

Further stratified analyses by academic year are presented in Table 3. Gender-related differences in the perception of dog welfare dimensions were identified in 2021/2022 and 2024/2025 academic years. In all cases, female students assigned higher scores than male students. Similarly, place of origin showed significant associations with several dimensions in selected academic years (2022/2023, 2023/2024, and 2025/2026), generally reproducing the pattern described in the overall analysis, with higher scores among students classified in the “other” category. An exception to this pattern was observed in the 2025/2026 cohort, where the highest scores were reported by students from Tenerife (*p* = 0.031). Overall, these findings suggest that the influence of demographic variables on the perception of dog welfare may vary across cohorts, reinforcing the presence of context-dependent and cohort-specific effects. These findings should be interpreted cautiously given the small size of certain subgroups.

## 4. Discussion

This study provides a comprehensive overview of veterinary students’ perceptions of key dog welfare issues across six academic years, revealing both consistent patterns and notable variability. Although several of the observed patterns are consistent with previous research in veterinary education and animal welfare perception [19,20], the present study contributes a novel multi-cohort, repeated cross-sectional perspective across six academic years within a single institutional context. To our knowledge, this is among the few studies to examine the stability and variability of these perceptions over such an extended temporal framework using a consistent and validated instrument. Overall, students assigned high importance to issues involving clear and acute suffering, such as abuse or active cruelty, while comparatively lower scores were observed for more complex or socially normalized conditions, including breed-related disorders. Although significant differences were identified across academic cohorts and several demographic variables, these variations did not follow a consistent temporal trend. Instead, the results suggest that students’ perceptions of dog welfare are shaped by a combination of cohort-specific and contextual factors, rather than a linear progression throughout training. Additionally, the influence of place of origin emerged as a relevant factor, particularly in relation to less visible or more interpretative dimensions of welfare. However, these findings should be interpreted considering several methodological considerations. The study is based on self-reported perceptions, which may not fully reflect actual knowledge or competencies and are therefore susceptible to response biases, including social desirability effects given the academic context of data collection. This is further compounded by the fact that, although participation was voluntary, its integration within course assessment may have introduced a degree of self-selection bias, as more engaged or motivated students may have been more likely to participate. In addition, the relatively small and heterogeneous “Other” origin group limits the interpretability of the analyses related to geographical background. Finally, as data were collected within a single institution, caution is warranted when generalizing the findings to other educational or cultural contexts.

Animal welfare is widely recognized as a complex and multidimensional construct that extends beyond the mere absence of disease or overt suffering, encompassing physical health, mental states, behavior, and environmental conditions [1,28]. Within this framework, the perception of welfare issues cannot be understood solely as a reflection of objective knowledge. Rather, it is shaped by a combination of cognitive, emotional, and contextual factors, including personal values, prior experiences, and social norms [1,18,23,24]. Consequently, awareness of a given welfare problem does not necessarily translate into its prioritization, highlighting a critical distinction between knowing and perceiving animal welfare concerns.

A comparison with the findings reported by Yeates and Main (2011), conducted among veterinary clinicians [19], reveals both convergences and notable divergences between students and professionals. In the present study, a higher proportion of participants rated obesity and chronic pain or poor mobility as highly important (51.6% and 67.5%, respectively) compared to the 27.6% and 46.4% reported by clinicians (*n* = 58). In contrast, breed-related conditions showed a different pattern: while 36.3% of students assigned a moderate importance score (2), only 19.3% of practicing clinicians in the study by Yeates did so, indicating that the clinical relevance of breed-associated disorders is generally recognized more fully through professional experience than during undergraduate training. Despite these differences, strong agreement was observed for issues involving overt and severe welfare compromise. In both studies, lack of treatment to prevent suffering, abuse or active cruelty, and malnutrition were consistently ranked among the most important concerns, with high proportions of respondents assigning the maximum score. This concordance reinforces the notion that clear and acute forms of suffering are readily recognized across different stages of veterinary training and professional practice. Comparison with the findings reported by Luño et al. for fifth-year veterinary students [20] reveals a close alignment with the present results. In both studies, abuse or active cruelty emerged as the issue with the highest mean score (3.9 in Luño et al. vs. 4.0 in the current series), while breed-related conditions consistently received the lowest mean scores (1.98 and 2.5, respectively). Similarly, ratings for dog behavioral problems were nearly identical across the two series (mean = 3.8). Minor divergences were observed for other items: obesity received a higher mean in the present study, whereas chronic pain or poor mobility scored slightly higher in the Luño et al. sample [21]. Overall, these similarities support the reproducibility of students’ perceptions across different cohorts and educational contexts, particularly for issues involving overt dog suffering versus more nuanced or breed-specific concerns.

These findings have important educational and practical implications [29]. The consistent prioritization of issues such as abuse or active cruelty, lack of treatment to prevent suffering, and malnutrition indicates that veterinary curricula effectively convey the ethical imperatives related to overt dog suffering [25]. Conversely, lower scores for breed-related conditions highlight areas where professional experience may be required to develop full awareness, suggesting that clinical exposure and case-based learning could enhance students’ understanding of more nuanced dog welfare concerns [6]. Importantly, the repeated cross-sectional comparison across six academic years revealed statistically significant differences in both individual items and aggregated welfare dimensions. This observation contributes to understand prior international studies reporting that year of study influences attitudes toward dog welfare, although in the present work such differences should be interpreted as differences between academic cohorts rather than developmental change [24,30,31]. Moreover, these results reinforce the notion that veterinary education is a dynamic and evolving process, influenced not only by curricular content but also by student demographics, prior experience, and sociocultural background [31,32]. In this context, the present study provides evidence supporting the value of repeated cross-sectional, cohort-based comparisons in veterinary curricula, highlighting the importance of tracking ethical reasoning and welfare perception over time and adapting teaching strategies accordingly. Additionally, the significant influence of students’ origin on both individual items and dimensions emphasizes that cultural and geographical factors may shape the development of dog welfare attitudes [31], which should be considered when designing educational interventions to ensure a comprehensive and inclusive approach to animal welfare training.

The greater sensitivity of female students toward animal welfare is well documented in the literature [24,33,34], and our findings corroborate this pattern. Similarly, students’ cultural and geographical background appears to influence welfare perceptions [35,36], partially explaining why participants originating from regions outside the two main islands of the archipelago tended to report higher scores across several items. To fully interpret these differences, it is important to consider additional demographic and experiential factors that may shape attitudes, such as hunting practices, or access to specific veterinary services [18,23,24]. Taken together, these results highlight that certain dog welfare issues warrant more targeted and proactive integration into the veterinary curriculum, as education plays a critical role in shaping ethical reasoning and perceptual sensitivity [37]. The present study underscores the value of systematically incorporating these topics into undergraduate training to foster a more comprehensive and nuanced understanding of animal welfare among future veterinarians.

These findings have broader implications for understanding the development of ethical decision-making in veterinary professionals. The variability observed in students’ prioritization of dog welfare issues across academic years, as well as according to gender and cultural background, suggests that ethical sensitivity is not uniform but evolves through a combination of formal education, experiential learning, and individual sociocultural context [38,39,40]. Ethical prioritizations appear to be shaped not only by curriculum content but also by the perspectives and experiences that students bring to their training. In this regard, the concept of the hidden curriculum may also be relevant, referring to the implicit values, norms, and behavioural models that students acquire through clinical environments, institutional culture, and observation of professional practice, which may subtly influence their perception of animal welfare priorities [41]. This supports the view that veterinary education should extend beyond the transmission of welfare science knowledge, incorporating structured opportunities for reflection, case-based learning, and engagement with ethical dilemmas [42,43]. Such a multidimensional approach may be essential to foster nuanced, context-aware ethical reasoning in future clinicians, ultimately supporting responsible clinical practice and effective advocacy for animal welfare. Although well explored in other healthy practitioners [44,45], this approach is less known among veterinary professionals.

This study has several strengths. First, this study provides a repeated cross-sectional assessment of veterinary students’ perceptions of dog welfare spanning six academic years, offering insights into variability across cohorts over time. Second, the use of a structured and previously published questionnaire [19,20] ensures comparability with prior research and supports the reliability of the findings. Third, the inclusion of multiple demographic variables, including gender, age, home setting, and place of origin, allows for a nuanced exploration of factors influencing students’ dog welfare perceptions. However, some limitations should be acknowledged. First, the study is based on self-reported perceptions, which may not fully reflect students’ actual knowledge or professional competencies regarding dog welfare, and are inherently susceptible to response biases. In particular, data collection within an academic context may have introduced social desirability bias, potentially leading to more favourable evaluations than those expressed in less evaluative settings. Second, although participation in the survey was voluntary, it was embedded within course assessment activities, which may have contributed to self-selection bias, as students more engaged or motivated may have been more likely to participate. The sample size per cohort is relatively small, with variable response rates (18.5–66.1%), raising additional concerns about selection bias. Additionally, differences observed between academic cohorts should be interpreted with caution, as cohorts may vary not only temporally but also in their demographic composition. Variations in factors such as gender distribution or place of origin across academic years were not controlled for in the analysis and may have contributed to some of the observed differences. Therefore, cohort-related findings cannot be attributed exclusively to temporal or educational effects and may reflect underlying demographic heterogeneity. Third, a large number of statistical comparisons were performed across individual items, composite dimensions, and demographic variables without formal adjustment for multiple testing. As a result, some statistically significant findings may be due to chance and should be interpreted with caution. Furthermore, some subgroup analyses were based on relatively small sample sizes, particularly within the ‘Other’ origin category and in year-specific stratified analyses. These small and uneven group sizes may reduce the robustness of statistically significant findings and warrant cautious interpretation of the present results. Finally, the study is limited to a single institution, and therefore the findings may not be generalizable to veterinary students in other universities or cultural contexts.

## 5. Conclusions

Veterinary students consistently recognize overt dog welfare issues, while sensitivity to breed-related conditions appears comparatively lower. Perceptions vary between cohorts across academic years, gender, and origin, although these differences should be interpreted with caution given the limited size of certain subgroups. Overall, the findings indicate that differences in dog welfare perceptions are associated with cohort characteristics and sociocultural background. These results highlight the importance of considering less visible and socially normalized welfare issues within veterinary education.

## Figures and Tables

**Table 1:** Association between demographic variables (gender, origin, and home setting) and veterinary students’ perception of the importance of different dog welfare issues (*n* = 157). Data are presented as mean Likert scores (0–4) ± standard deviation (SD). Statistically significant results are highlighted in bold.

	Gender ^1^		Origin ^2^			Home Setting ^1^	
Issue	Female (*n* = 119)	Male (*n* = 38)	Gran Canaria (*n* = 101)	Tenerife (*n* = 27)	Others ^3^ (*n* = 24)	Rural (*n* = 77)	Urban (*n* = 69)
Obesity	3.4 ± 0.7	3.2 ± 0.9	3.4 ± 0.7	3.3 ± 0.8	3.6 ± 0.6	3.4 ± 0.7	3.4 ± 0.8
*p* value	0.248		0.472			0.658	
Chronic pain or poor mobility	3.7 ± 0.6	3.6 ± 0.7	3.7 ± 0.5	3.4 ± 0.9	3.8 ± 0.4	3.7 ± 0.5	3.6 ± 0.7
*p* value	0.703		0.139			0.873	
Breed-related conditions	2.5 ± 0.9	2.6 ± 0.9	2.5 ± 0.9	2.3 ± 0.9	3.0 ± 0.9	2.5 ± 0.9	2.5 ± 0.9
*p* value	0.933		**0.038**			0.661	
Behavioral problems	2.8 ± 0.9	2.9 ± 0.8	2.8 ± 0.9	2.6 ± 1.1	3.2 ± 0.7	2.8 ± 0.9	2.7 ± 0.9
*p* value	0.883		**0.033**			0.650	
Lack of treatment *	3.8 ± 0.5	3.9 ± 0.3	3.9 ± 0.3	3.7 ± 0.8	3.9 ± 0.3	3.9 ± 0.3	3.8 ± 0.6
*p* value	0.352		0.831			0.130	
Lack of sufficient exercise or space	3.6 ± 0.6	3.4 ± 0.7	3.4 ± 0.6	3.6 ± 0.7	3.8 ± 0.5	3.4 ± 0.7	3.6 ± 0.6
*p* value	0.204		**0.010**			0.182	
Lack of sufficient mental stimulation	3.3 ± 0.7	3.4 ± 0.6	3.2 ± 0.7	3.2 ± 0.8	3.6 ± 0.5	3.3 ± 0.7	3.3 ± 0.7
*p* value	0.723		0.085			0.974	
Lack of routine preventive veterinary care	3.4 ± 0.8	3.4 ± 0.7	3.4 ± 0.8	3.2 ± 0.9	3.6 ± 0.6	3.4 ± 0.8	3.4 ± 0.8
*p* value	0.707		0.157			0.653	
Abuse or active cruelty	3.9 ± 0.4	3.9 ± 0.1	3.9 ± 0.1	3.8 ± 0.8	3.9 ± 0.1	3.9 ± 0.1	3.9 ± 0.5
*p* value	0.423		0.448			0.134	
Malnutrition	3.9 ± 0.4	3.2 ± 0.2	3.9 ± 0.2	3.8 ± 0.8	3.9 ± 0.2	3.9 ± 0.3	3.9 ± 0.5
*p* value	0.652		0.565			0.498	
Lack of sufficient company	3.4 ± 0.7	3.2 ± 0.9	3.3 ± 0.7	3.3 ± 0.9	3.7 ± 0.5	3.4 ± 0.7	3.3 ± 0.8
*p* value	0.289		**0.026**			0.597	
Lack of shelter	3.6 ± 0.7	3.4 ± 0.8	3.5 ± 0.7	3.7 ± 0.9	3.9 ± 0.3	3.7 ± 0.6	3.6 ± 0.8
*p* value	0.114		**0.017**			0.443	

* Including euthanasia for suffering. ^1^ Mann–Whitney U test. ^2^ Kruskal–Wallis test. ^3^ Exchange students with unavailable origin.

**Table 2:** Association between demographic variables (gender, origin, and home setting) and veterinary students’ perception of the importance of different dog welfare dimensions (*n* = 157). Data are presented as mean ± standard deviation (SD). Statistically significant results are highlighted in bold.

	Gender *		Origin **			Home Setting *	
Dimension	Female (*n* = 119)	Male (*n* = 38)	Gran Canaria (*n* = 101)	Tenerife (*n* = 27)	Others *** (*n* = 24)	Rural (*n* = 77)	Urban (*n* = 69)
Physical welfare ^1^	7.6 ± 0.9	7.6 ± 0.7	7.6 ± 0.6	7.2 ± 1.6	7.8 ± 0.5	7.6 ± 0.6	7.5 ± 1.1
*p* value	0.820		0.104			0.979	
Mental welfare ^2^	9.5 ± 1.9	9.4 ± 1.6	9.3 ± 1.7	9.1 ± 2.4	10.6 ± 1.3	9.5 ± 1.7	9.3 ± 2.0
*p* value	0.694		**0.005**			0.811	
Environmental structural welfare ^3^	7.2 ± 1.1	6.8 ± 1.1	6.9 ± 1.1	7.3 ± 1.5	7.7 ± 0.6	7.1 ± 1.2	7.1 ± 1.2
*p* value	**0.037**		**0.002**			0.690	
Socially normalized issues ^4^	5.9 ± 1.3	5.7 ± 1.7	5.9 ± 1.3	5.7 ± 1.6	6.5 ± 1.3	5.9 ± 1.3	5.9 ± 1.5
*p* value	0.678		0.058			0.514	
Malpractice ^5^	11.2 ± 1.3	11.3 ± 0.8	11.2 ± 0.9	10.8 ± 2.2	11.5 ± 0.7	11.3 ± 1.0	11.1 ± 1.5
*p* value	0.882		0.198			0.764	
Total ^6^	41.4 ± 5.3	41.0 ± 4.8	41.0 ± 4.3	40.1 ± 8.2	44.0 ± 2.9	41.3 ± 4.2	41.0 ± 6.1
*p* value	0.411		**0.008**			0.738	

^1^ Sum of items 2 (Chronic pain or limited mobility) and 10 (Malnutrition). Maximum score: 8. ^2^ Sum of items 4 (Behavioral problems), 7 (Lack of sufficient mental stimulation), and 11 (Lack of company). Maximum score: 12. ^3^ Sum of items 6 (Lack of sufficient exercise or space) and 12 (Lack of shelter). Maximum score: 8. ^4^ Sum of items 1 (Obesity) and 3 (Breed-related conditions). Maximum score: 8. ^5^ Sum of items 5 (Lack of treatment to avoid suffering (including euthanasia), 8 (Lack of routine preventive veterinary care) and 9 (Abuse or active cruelty). Maximum score: 12. ^6^ Sum of all items. Maximum score: 48. * Mann–Whitney U test. ** Kruskal–Wallis test. *** Exchange students with unavailable origin.

**Table 3:** Association between demographic variables and veterinary students’ perception of the importance of different dog welfare dimensions by academic year. Data are presented as mean ± standard deviation (SD). Only statistically significant results are included.

	Gender *		Origin **		
Academic Year/Dimension	Female	Male	Gran Canaria	Tenerife	Others ***
2021/2022	(*n* = 20)	(*n* = 6)	(*n* = 19)	(*n* = 5)	(*n* = 1)
Environmental structural welfare	6.85 ± 1.3	5.7 ± 1.2	—	—	—
*p* value	0.037		n.s.		
2022/2023	(*n* = 20)	(*n* = 9)	(*n* = 17)	(*n* = 5)	(*n* = 5)
Physical welfare	—	—	7.9 ± 0.2	5.8 ± 3.3	7.8 ± 0.4
*p* value	n.s.		0.018		
Mental welfare	—	—	9.2 ± 1.7	6.4 ± 3.1	10.8 ± 1.1
*p* value	n.s.		0.009		
Total	—	—	41.3 ± 4.5	32.4 ± 15.4	44.6 ± 3.3
*p* value	n.s.		0.035		
2023/2024	(*n* = 20)	(*n* = 6)	(*n* = 14)	(*n* = 5)	(*n* = 6)
Socially normalized issues	—	—	5.6 ± 1.3	6.2 ± 1.3	7.7 ± 0.5
*p* value	n.s.		0.005		
2024/2025	(*n* = 31)	(*n* = 8)	(*n* = 27)	(*n* = 5)	(*n* = 7)
Mental welfare	10.0 ± 1.3	8.9 ± 0.8	—	—	—
*p* value	0.021		n.s.		
Total	42.4 ± 3.6	39.4 ± 3.4	—	—	—
*p* value	0.019		n.s.		
2025/2026	(*n* = 21)	(*n* = 4)	(*n* = 16)	(*n* = 5)	(*n* = 4)
Socially normalized issues	—	—	6.7 ± 1.0	7.0 ± 0.7	5.3 ± 0.5
*p* value	n.s.		0.031		

Abbreviation: n.s., non-significant. * Mann–Whitney U test. ** Kruskal–Wallis test. *** Exchange students with unavailable origin.

## Data Availability

The data presented in this study are available from the corresponding author upon reasonable request.

## Supplementary Materials

The following supporting information can be downloaded at: https://www.mdpi.com/article/10.3390/ani16091385/s1, Table S1: Demographic characteristics of the study population by academic year. The table presents the number of respondents relative to the total number of enrolled students for each academic year, along with descriptive statistics for age (mean ± standard deviation (SD)) and frequency distributions for gender, origin (Gran Canaria, Tenerife, and others), and habitat (rural vs. urban); Table S2: Importance (*n*, (%), median and mean ± standard deviation (SD)) for each issue in the wholes series (*n* = 157).
