# Supplementary material for: Perception of Dog Welfare in Veterinary Students: A Six-Year Study of Ethical Priorities, Cohort Variation, and Influencing Factors
Source: Animals (Basel). 2026 May 1;16(9):1385. doi: 10.3390/ani16091385 (PMC13162677; doi:10.3390/ani16091385)
Supplement: Supplementary file 1 [file animals-16-01385-s001.zip › animals-4254128-supplementary/Supplementary Table S2.pdf]

Supplementary Table S2. Importance (n, (%), median and mean ± standard deviation (SD)) for each issue in the wholes series (n = 157).

	Importance for individual dog						
	0	1	2	3	4	Median	Mean ± SD
Obesity	0	4 (2.5)	10 (6.4)	62 (39.5)	81 (51.6)	4	3.4 ± 0.7
Chronic pain or poor mobility	1 (0.6)	1 (0.6)	2 (1.3)	47 (27.9)	106 (67.5)	4	3.6 ± 0.6
Breed-related conditions	3 (1.9)	17 (10.8)	57 (36.3)	56 (35.7)	24 (15.3)	3	**2.5 ± 0.9**
Behavioural problems	2 (1.3)	13 (8.3)	33 (21.0)	73 (46.5)	36 (22.9)	3	2.8 ± 0.9
Lack of treatment*	1 (0.6)	0	1 (0.6)	17 (10.8)	138 (87.9)	4	3.9 ± 0.5
Lack of sufficient exercise or space	0	3 (1.9)	4 (2.5)	59 (37.6)	91 (58.0)	4	3.5 ± 0.6
Lack of sufficient mental stimulation	0	2 (1.3)	13 (8.3)	76 (48.9)	66 (42.0)	3	3.3 ± 0.7
Lack of routine preventive veterinary care	0	5 (3.2)	15 (9.6)	51 (32.5)	86 (54.8)	4	3.4 ± 0.8
Abuse or active cruelty	1 (0.6)	0	0	1 (0.6)	155 (98.7)	4	**4.0 ± 0.3**
Malnutrition	1 (0.6)	0	1 (0.6)	4 (2.5)	151 (96.2)	4	3.9 ± 0.4
Lack of sufficient company	2 (1.3)	1 (0.6)	12 (7.6)	66 (42.0)	76 (48.4)	3	3.4 ± 0.8
Lack of shelter	1 (0.6)	2 (1.3)	9 (5.7)	35 (22.3)	110 (70.1)	4	3.6 ± 0.7

* Including euthanasia for suffering.
